# Review of the Palaearctic (and Oriental) *Allurus* (Braconidae, Euphorinae) based on material from Sweden

**DOI:** 10.3897/BDJ.4.e7853

**Published:** 2016-03-09

**Authors:** Julia Stigenberg, Kees Van Achterberg

**Affiliations:** ‡ Swedish Museum of Natural History, Stockholm, Sweden; § Department of Terrestrial Zoology, Naturalis Biodiversity Center, Leiden, Netherlands; | College of Life Sciences, Northwest University, Xi’an, China

**Keywords:** Braconidae, Euphorinae, *
Allurus
*, parasitic wasp, DNA

## Abstract

**Background:**

The tribe Centistini includes three genera, *Allurus*, *Centistes* and *Centistoides* (Stigenberg et al. 2015). They are solitary endoparasitoids of adults and final instar larvae of beetles from the family Curculionidae (Jackson 1920, Aeschlimann 1980, Tobias 1986)

**New information:**

In this paper we present a key, molecular data (standard DNA barcode, CO1) and images of the two species of *Allurus* occurring in the Western Palaearctic. A third Oriental species described from China (Taiwan) is also included in the key. *Allurus* is a Holarctic genus with three known species (*A. choui*, *A. lituratus*, *A. muricatus*). Our sequence data confirms that *A. muricatus* and *A. lituratus* are two distinct and separate species and this paper points out good and easy characters to separate them.

## Introduction

**Figure 1.**
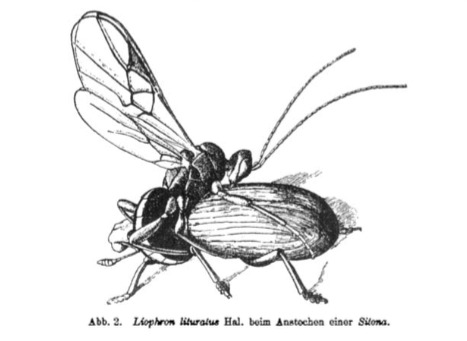
Illustration in Brammanis 1932 of a *A. lituratus* attacking its prey, a *Sitona* beetle.

The genus *Allurus* Förster contains fairly large euphorine wasps of 3-4 mm body length. It is a Holarctic genus with extralimital species in Taiwan. In this paper we present a review of the species of *Allurus* based on morphological study of the types and morphological and molecular study of the material available at NHRS. The *Allurus* are rare, but as they parasitize on the very common coleopteran genus *Sitona* (Curculionidae), they probably are more common than we think. Brammanis (1932) writes that this parasite grabs its prey with a sudden jump, mounts the beetle transversely at the front and pierce the ovipositor in between the pro- and mesothorax Fig. 1. For the beetle it is impossible to escape from this situation. The pupation takes place in a cocoon on the ground. The cocoon is 3 mm in length, pale cream colour, covered with some loose flocculence and constructed among moss on ground (Jackson 1920.) The pupal stage lasts for 12-13 days.

## Materials and methods

All specimens in the collections at the Swedish Museum of Natural History were collected with Malaise Traps. Five specimens of *A. lituratus* were collected within the Swedish Malaise Trap Project (SMTP). Two specimens were collected by A. Ohlsson in 2011. The single specimen of *A. muricatus* was collected in a allotment garden by T. Malm and M. Malm in 2012. Terminology used for morphological and wing structures follow Wharton et al. (1997)and Goulet and Hubert (1993) (in parenthesis). Institutional abbreviations: National Museum Ireland (Dublin, Irland) – NMI, Taiwan Agriculture Research Institute (Wufeng, Taiwan) – TARI, Zoological Institute, Russian Academy of Scienses (St. Petersburg, Russia) – ZIN, Swedish Museum of Natural History (Stockholm, Sweden) – NHRS, Swedish Malaise Trap Project – SMTP. For molecular methods regarding the mitochondrial marker CO1 see Stigenberg et al. (2015). Sequences were assembled, edited and imaged using Geneious version 8.1 created by Biomatters. Voseq 1.7.3 (Peña and Malm 2012) database was used for storing voucher and DNA sequence data. Sequences are deposited at GenBank under the accession numbers: KU521563 to 65, with an additional sequence KJ591423 from an earlier publication (Stigenberg et al. 2015). Images were taken using Canon EOS D50 with a MP-E 65 mm lens. The images were stacked using Zerene Stacker software.

## Taxon treatments

### Allurus

Förster 1863

Allurus Förster 1863
Allurus Ancylus muricatus Haliday 1833Haliday 1833

#### Diagnosis

The genus *Allurus* is diagnosed by having bifurcate claws, hind coxa with more or less developed ventral denticle, first metasomal tergite sessile, broad and strongly curved ovipositor, first subdiscal cell (2Cu) open, vein 2–1A (A) on fore wing not reaching wing vein 2Cua (Cu), laterope usually hardly visible.

### Allurus choui

Belokobylskij 2004

Allurus choui Belokobylskij 2004

#### Diagnosis

Precoxal sulcus absent. Notauli shallow and almost smooth. Marginal cell of fore wing distinctly shortened; metacarpus 0.9 times as long as pterostigma. Propodeum without transverse carina. Frons almost entirely sculptured. Body length 2.4–4.2 mm.

#### Distribution

Oriental (China, Taiwan).

#### Notes

Holotype: ♀, Taiwan, “C. Taiwan: Tsuifeng, 2300 m, Nantou Hsien, V.1984, K.S. Lin & K.C. Chou, Malaise trap” (TARI).

### Allurus lituratus

Haliday 1835

Leiophron (Ancylus) lituratus Haliday 1835
Leiophron armatus var. 1 Wesmael 1835, syn by Reinhard 1862
Allurus lituratus Type material: Lectotype designated by van Achterberg 1997. [NMI]

#### Diagnosis

Antenna with 30-33 segments, clypeus smoothly continuing from face, rather densely setose. Hind coxa with only slightly protruding denticle apico-ventrally, second metasomal sternite without denticles, if with denticles then on 4^th^ sternite, metasoma ventrally dense setose, first metasomal tergite only slightly longer than its apically width.

#### Distribution

Eastern Palaearctic, Western Palaearctic, Oriental and Nearctic.

#### Notes

**Studied material:** 1 ♀ Sweden, Sk, Klippans kommun, Skäralid, valley below northern Lierna. Rich beech forest. 11.vi–03.vii.2004. Trap ID 37, Coll ID 832. Leg. SMTP/NHRS. DNA voucher: JS10_00410. 1 ♀ Sweden, Sk, Höganäs kommun, Kullabergs naturreservat, between Hjortstugan and Ransvik, Oak forest in southern slope. 09.viii–20.ix.2005. Trap ID 1004, Coll ID 1898. Leg. SMTP/NHRS. DNA voucher: JS10_00409. GenBank accession: KJ591423. 1 ♀ Sweden, Sö, Haninge kommun, Tyresta Urskogsslingan. Flat-rock and pine forest. 20.vii–11.viii.2004. Trap ID 3, Coll ID 799. Leg. SMTP/NHRS. DNA voucher: JS10_00408. GenBank accession: KU521565. 1 ♀ Sweden, Sm, Älmhults kommun, Stenbrohult, Djäknabygds bokbacke, Heath with old beeches. 26.vi–15.vii.2003. Trap ID 24, Coll ID 816. Leg. SMTP/NHRS. DNA voucher: JS10_00407. 1 ♀ Sweden, Sm, Älmhults kommun, Stenbrohult, Djäknabygds bokbacke, Heath with old beeches. 20.vii–5.ix.2005. Trap ID 24, Coll ID 1674. Leg. SMTP/NHRS. DNA voucher: JS10_00406. GenBank accession: KU521564. 2 ♀ SWE. SK. Klippan, Söderåsens NP. Skärsån. Malaise Trap. N56°02.225', E13°14,377'. 18.vi.2011–02.vii., 35 moh. Loc023-06. Leg. A. Ohlsson. All specimens are deposited at NHRS.

### Allurus muricatus

Haliday 1833

Ancylus muricatus Haliday 1833
Leiophron (Ancylus) muricatus Haliday 1835
Leiophron armatus var. 2 Wesmael 1835 syn. by Curtis 1837
Allurus muricatus var. *nigra*Lyle 1926 (= Wesmael's var. 2). Lyle became author by referring to var. 2 as (var. *nigra* Wesm.).
Allurus muricatus Type specimen: Lectotype designated by van Achterberg 1997. [NMI]

#### Diagnosis

Antenna with 30 segments, clypeus distinctly differentiated from face and sparsely setose. Hind coxa with a thumb-like denticle apico-ventrally, second metasomal sternite with two distinctly protruding denticles, metasoma ventrally sparse setose, first metasomal tergite as broad apically as long.

#### Distribution

Eastern Palaearctic and Western Palaearctic.

#### Notes

**Figure 3.**
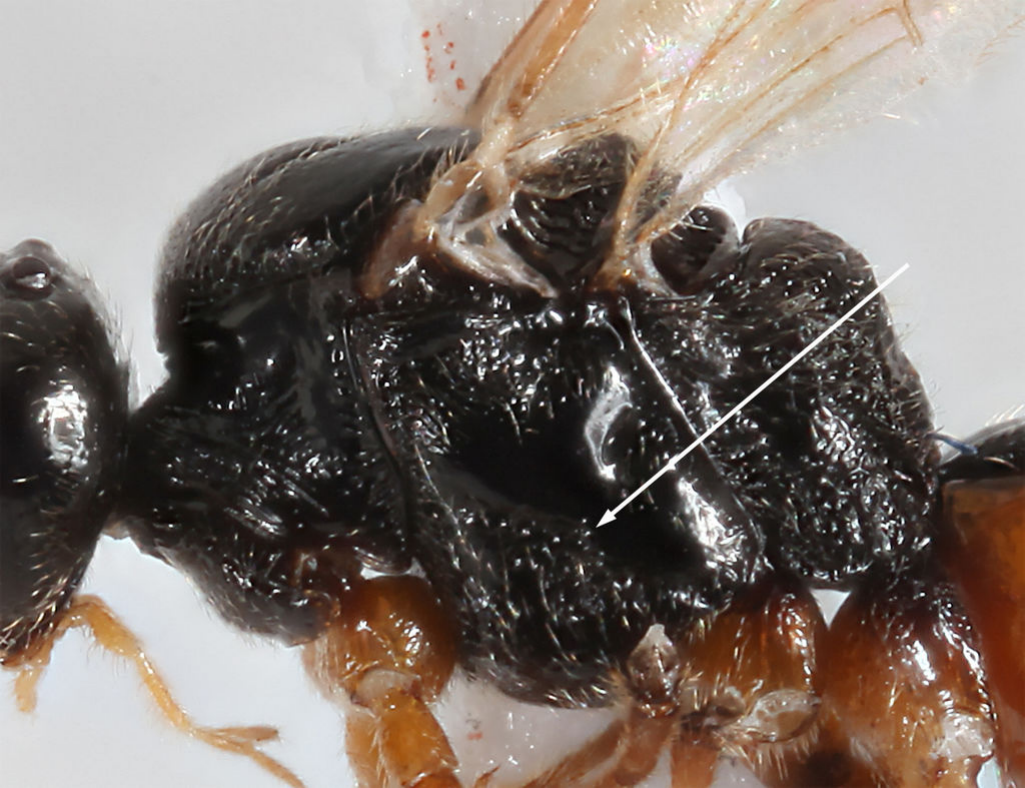
Lateral view of mesosoma, the arrow indicating precoxal sulcus of *A. muricatus*. Image of voucher specimen JS10_00405.

**Figure 5.**
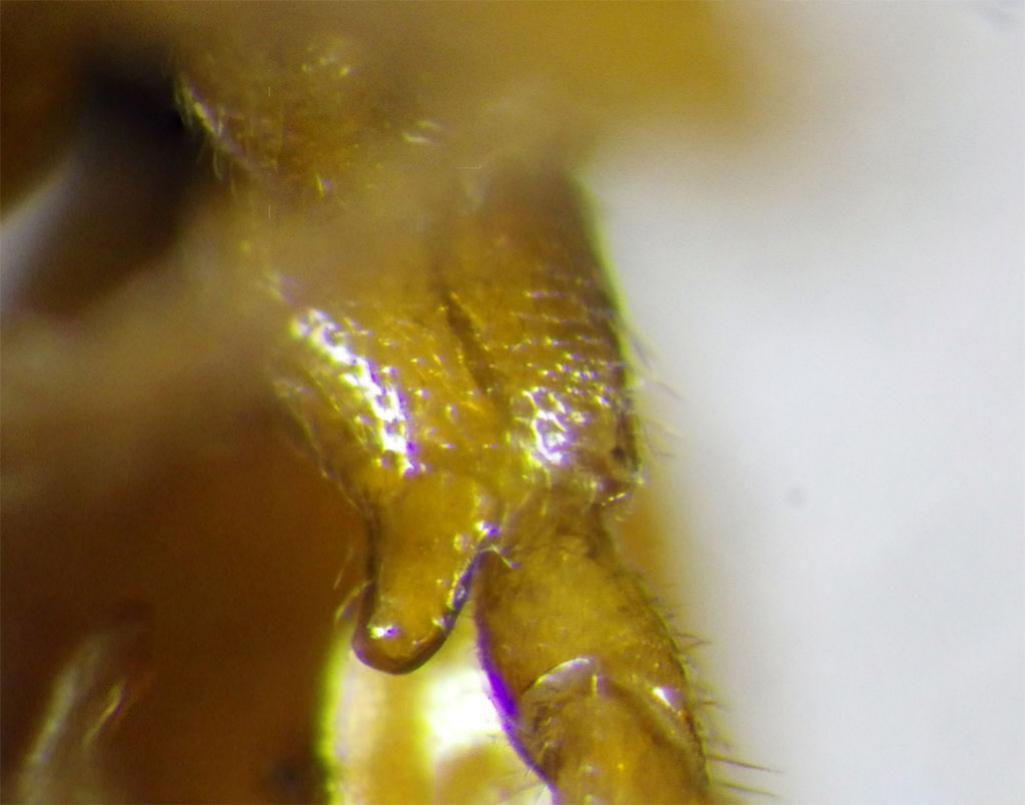
Ventral view of hind coxa showing apical denticle of *A. muricatus*. Image of voucher specimen JS10_00405.

**Figure 7.**
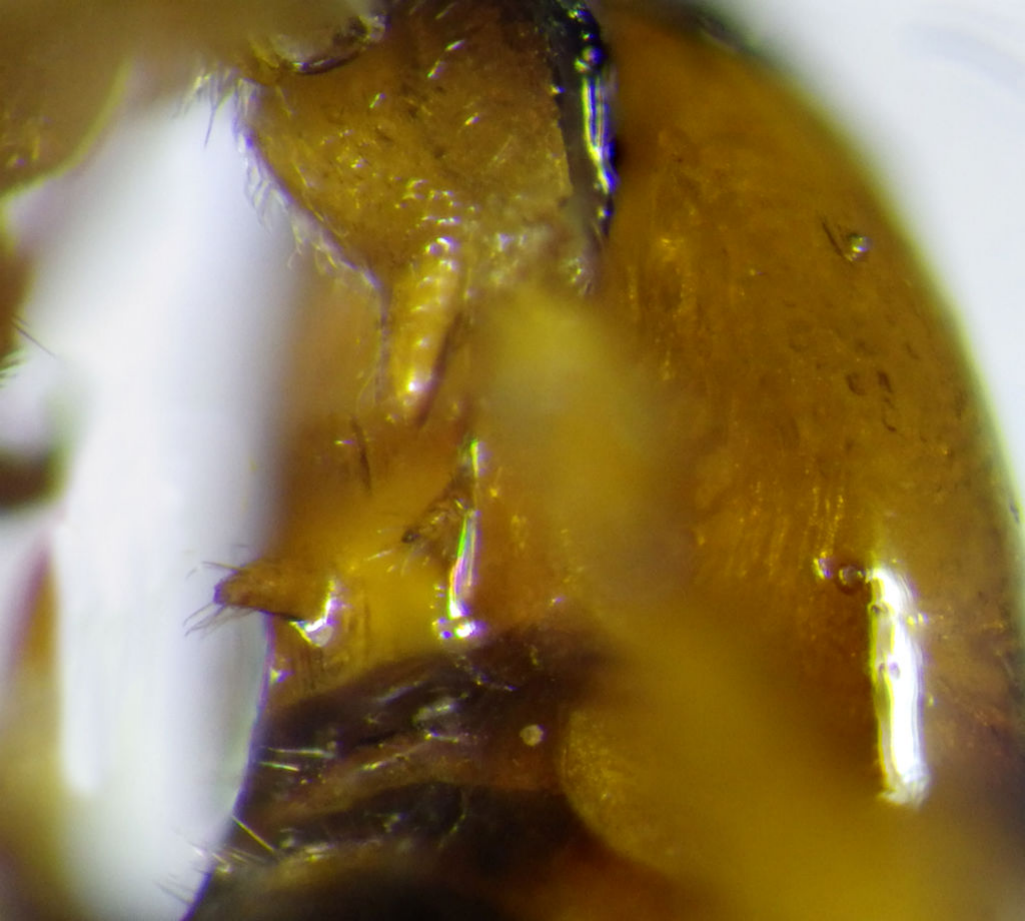
Ventral view of the second metasomal sternite with two distinctly protruding denticles of *A. muricatus*. Image of voucher specimen JS10_00405.

**Figure 9.**
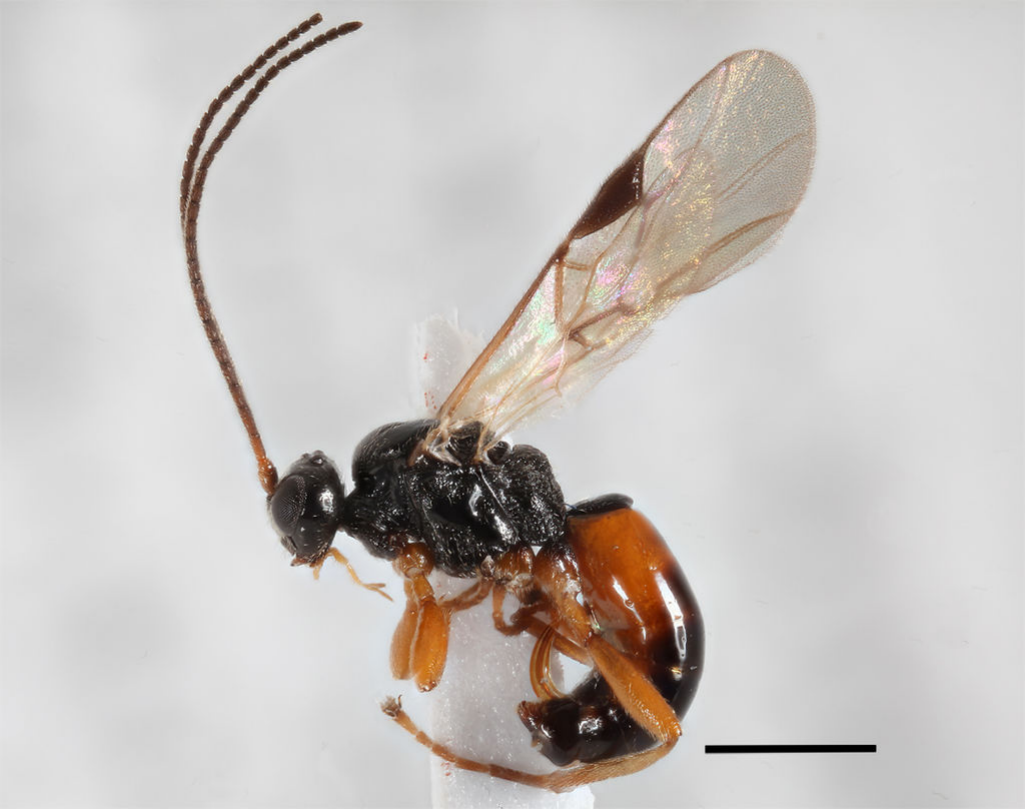
Lateral habitus of *A. muricatus*. Image of voucher specimen JS10_00405. The scale bar equals 1 mm.

**Studied material:** 1 ♀ Sweden, Sö, Stockholm, Enskede Gårds kolonilottsförening, N59°17'19.58", E18°03'54.63" Malaisetrap, 10-25.vii.20012, leg. T. Malm & M. Malm. DNA voucher: JS10_00405. GenBank accession: KU521563. The specimen is deposited at NHRS. All photos of *A. muricatus* belong to this specimen (Figs 3, 5, 7, 9).

## Identification Keys

### Key to the species of *Allurus*

**Table:** 

1	Precoxal sulcus absent Fig. 2, notauli shallow and almost smooth	*Allurus choui*
–	Precoxal sulcus present (Figs 3, 4), notauli distinct	2
2	Hind coxae with distinct denticle apico- ventrally Fig. 5, second metasomal sternite with two distinctly protruding denticles Fig. 7, metasoma ventrally without dense setae, clypeus distinctly emerging, clypeus sparsely setose, first metasomal tergite as broad apically as long, mesosternum punctulate, posterior face of propodeum gradually sloping, habitus as Fig. 9.	*Allurus muricatus*
–	Hind coxae with less developed and only slightly protruding denticle apico- ventrally Fig. 6, second metasomal sternite without denticles, if present then on 4^th^ sternite Fig. 8, metasoma ventrally densely setose, clypeus smoothly continuing from face, clypeus rather densely setose, first metasomal tergite only slightly longer than apically broad, mesosternum punctate, posterior face of propodeum comparatively steep, habitus as Fig. 10.	*Allurus lituratus*

## Analysis

**Figure 11.**
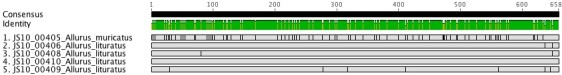
Alignment showing the disagreements between sequences of the *Allurus* specimens.

The analysis of the sequences revealed a nucleotide difference of 12.5% (82 bases differing) between the sequence of *A. muricatus* and the four sequences of *A. lituratus*. The intraspecific variation among *A. lituratus* was within 1.5% difference, with the specimen JS10_00409 differing from the other *A. lituratus* with 7-9 bases. There was no specific part of the sequences where the nucleotid disagreements between the two species were aggregated, they were spread all over the 658 nucleotide bases (Fig. 11).

## Discussion

The SMTP has collected insects since 2003 through out Sweden and that the result of those trappings should be only a handful of *Allurus* specimens is rather strange, that is, in relation to the common occurrence of the host genus. The *Sitona* are rather common all over Sweden and found even in Jukkasjärvi, 200 km north of the Arctic circle (SLU, Swedish University of Agriculture 2015). The Swedish *Allurus* speciemens were only collected in the following southern provinces: Skåne, Småland and Sörmland. Hopefully this paper will increase the interest and effort for amateurs to collect and identify braconid wasps.

## Supplementary Material

XML Treatment for Allurus

XML Treatment for Allurus choui

XML Treatment for Allurus lituratus

XML Treatment for Allurus muricatus

## Figures and Tables

**Figure 2.**
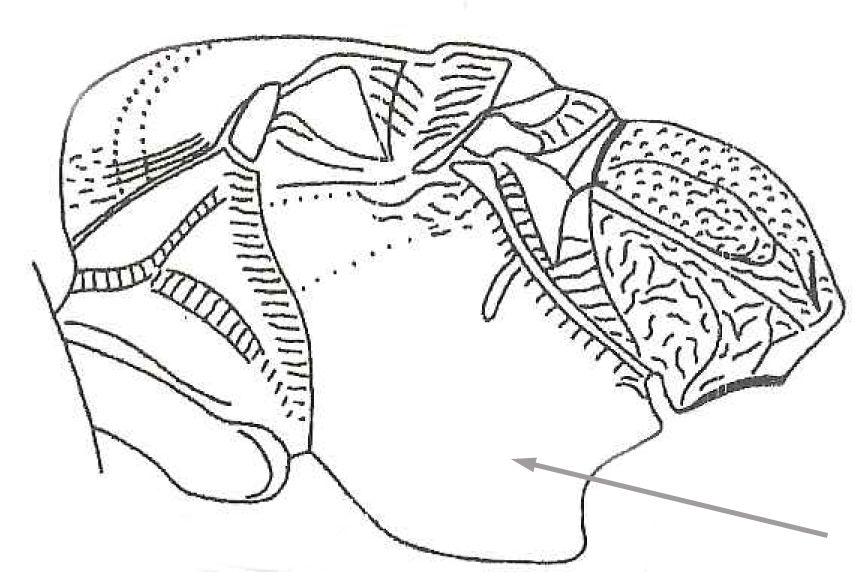
Lateral view of mesosoma, arrow showing absence of precoxal sulcus of *A. choui*. Illustration from Belokobylskij 2004.

**Figure 4.**
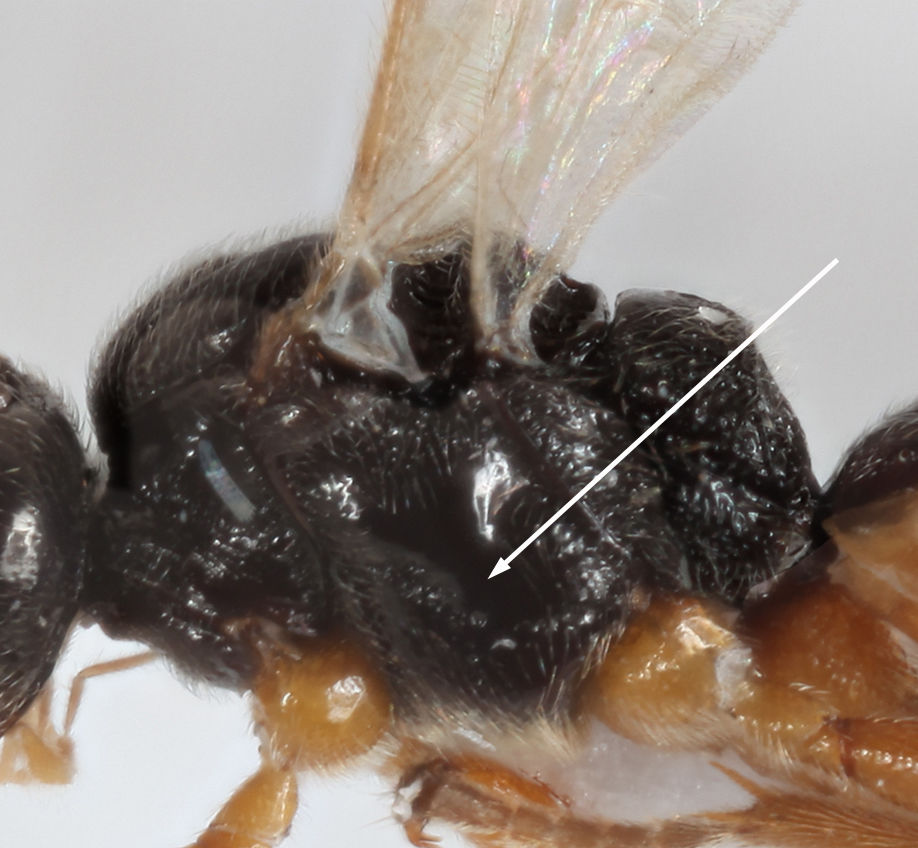
Lateral view of mesosoma, the arrow indicating precoxal sulcus of *A. lituratus*. Image of voucher specimen JS10_00406.

**Figure 6.**
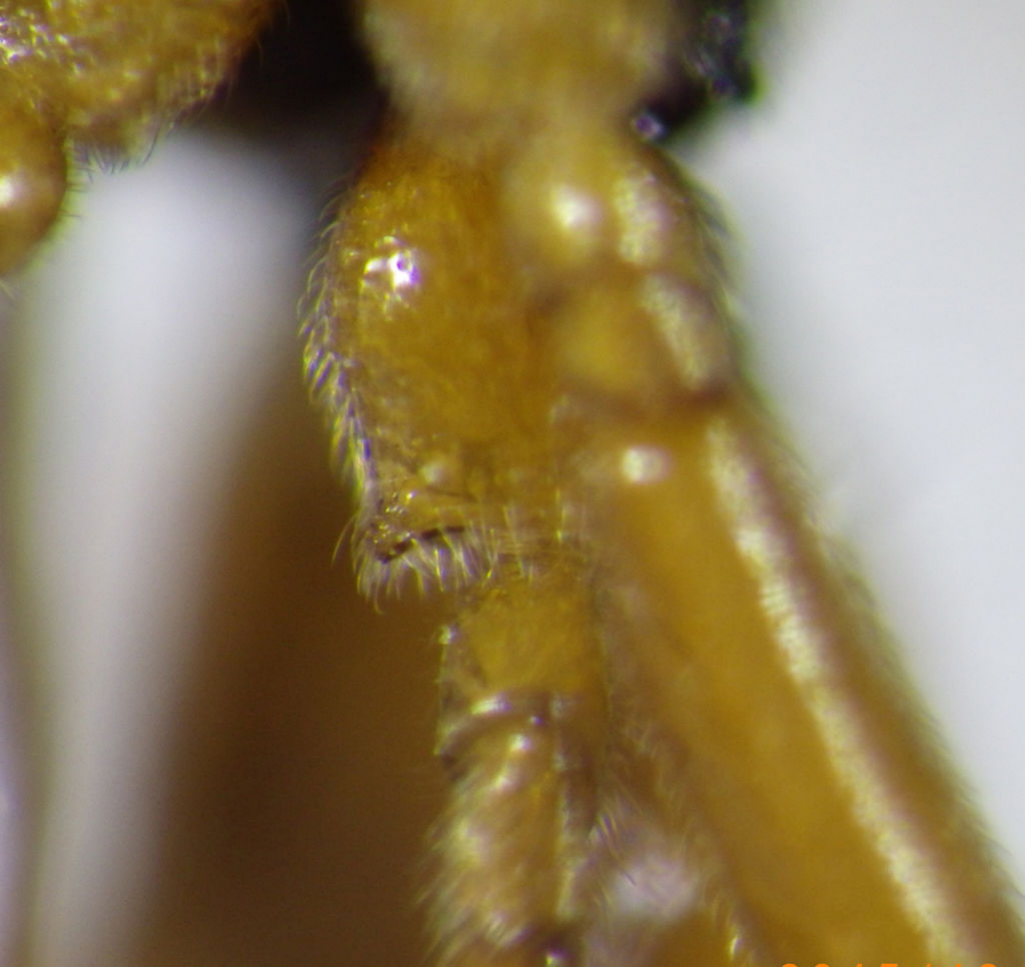
Ventral view of hind coxa showing apical denticle of *A. lituratus*. Image of voucher specimen JS10_00407.

**Figure 8.**
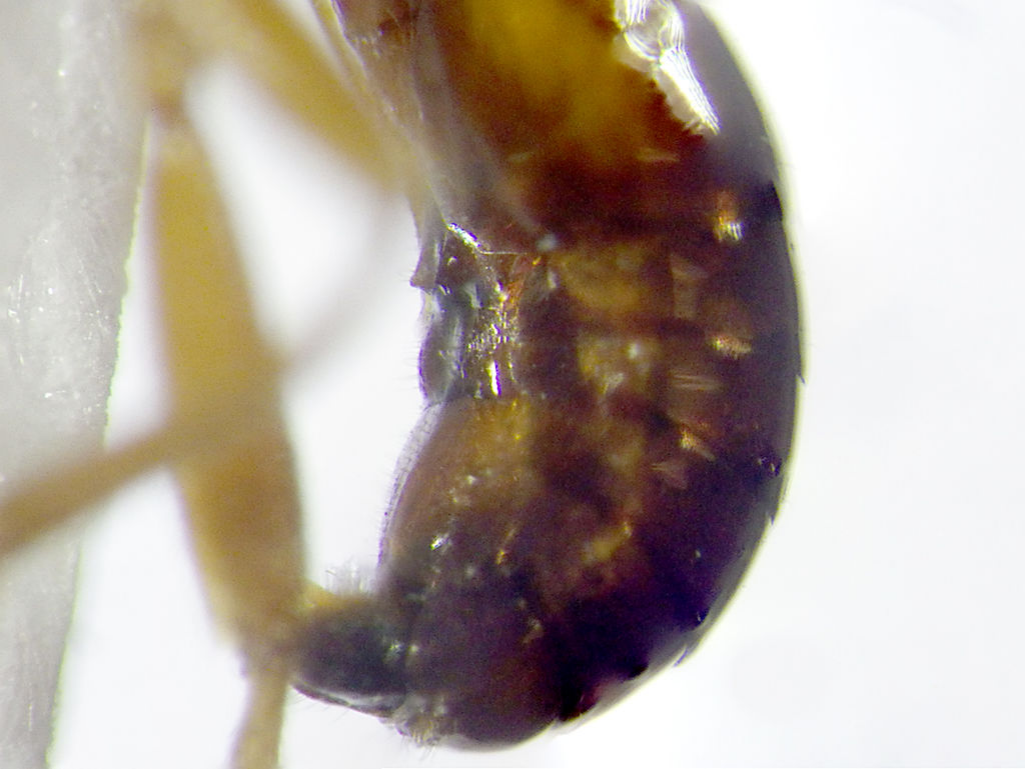
Ventral view of the fourth metasomal sternite with two slightly protruding denticles of *A. lituratus*. Image of voucher specimen JS10_00407.

**Figure 10.**
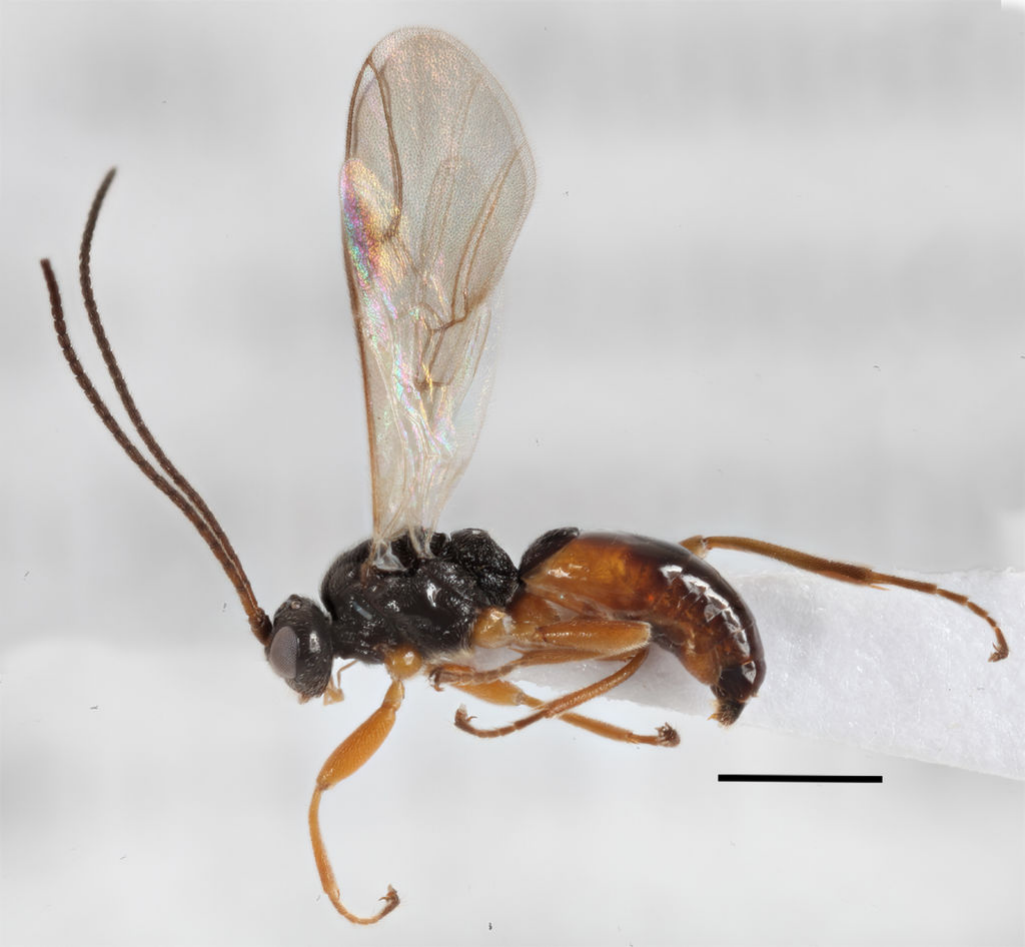
Lateral habitus of *A. lituratus*. Image of voucher specimen JS10_00406. The scale bar equals 1 mm.
